# Do newly marketed generic medicines expand markets using descriptive time series analysis and mixed logit models? Korea as an exemplar and its implications

**DOI:** 10.1186/s12913-016-1356-z

**Published:** 2016-04-14

**Authors:** Hye-Young Kwon, Brian Godman

**Affiliations:** Division of Biomedicine & Public Health, Mokwon University, Daejeon, 35349 South Korea; Division of Clinical Pharmacology, Department of Laboratory Medicine, Karolinska Institute, Karolinska University Hospital Huddinge, SE-141 86 Stockholm, Sweden; Strathclyde Institute of Pharmacy and Biomedical Sciences, University of Strathclyde, Glasgow, G4 0RE UK

**Keywords:** Generics, Market expansion, Korea, Statins, Health policy

## Abstract

**Background:**

There have been concerns in Korea that the availability of cheaper generics can appreciably increase prescribed volumes thereby negating their beneficial effects on overall pharmaceutical expenditure.

Consequently, this study aimed to explore market changes after newly entered generics including market expansion and substitution effects, and to examine factors contributing to the prescribing of newly entered generics using atorvastatin as an exemplar. This is because previous studies have shown market expansion had occurred following generic atorvastatin.

**Methods:**

Explore market expansion effects by extracting all statins users from July 2008 to June 2010 from the nationally representative dataset of 2008, combined with the National Health Insurance Claims data, with atorvastatin’s patent expiring in July 2008. The data consisted of medical visit episodes of patients who had been prescribed statins at least once during the observational period. Patients who had been prescribed any statin before the observation period were classified as the previously treated group and those who had not as the newly treated group. Descriptive time series analysis was conducted and the mixed logit model applied to understand factors contributing to generic atorvastatin prescriptions.

**Results:**

Market expansion was observed after generic atorvastatin availability with an appreciable increase in number of newly treated patients, whereas substitution effect was found among previously treated patients. Newly treated patients tended to get significantly lower daily doses (*p* < 0.0001). According to the mixed logistic analysis, newly treated patients were more likely to be prescribed generic atorvastatin (OR = 2.58; 95 % CI, 2.05–3.26) than their counterparts. Clinicians and secondary hospitals were also key drivers of generic atorvastatin (ORs were 10.41 and 9.81, respectively).

**Conclusions:**

Newly marketed generic statins in Korea resulted in an expanding market by substantially increasing the number of new patients with clinics and hospitals appreciably using newly marketed generics. However lower doses of statins were prescribed. Policy makers do recognize that generic availability can save costs so should be encouraged. However, this is a concern when generic availability appreciably expands the market, potentially increasing the financial burden. This needs to be addressed. Additionally in Korea, the quality of prescribing should be monitored, especially focusing on clinics and secondary hospitals.

## Background

Many countries are pursuing policies of encouraging the utilization of less costly generic medicines to cut spending and ensure efficient resource distribution. It is generally believed that generic medicines are less expensive copies of originators with the same therapeutic effects and the potential for appreciable savings [1–11]. Considerable resources can also be saved without compromising patient care by encouraging the prescribing of low costs generics versus more expensive patented products in classes where all products are seen as essentially similar at therapeutic doses. These include the proton pump inhibitors (PPIs), renin-angiotensin inhibitors and statins [6, 12–16]. However, the actual cost-savings arising from increased utilization of generics versus originators vary from country to country depending on the pricing policies for generics in that country as well as the extent of demand-side measures encouraging their prescribing [3–8]. The prerequisite for yielding appreciable cost-savings is that originators are substituted by less costly generics and the overall volume of consumption does not undergo significant changes.

However, according to the report of an analysis conducted by Kanovas et al. (2008), no such savings were realized in the seven key OECD countries despite low prices and higher generic penetration [9]. This may be due to different policies across countries enhancing the prescribing of generics versus patented products in a class, with pharmaceutical companies continuing to promote their patented medicines, which can be up to 20 to 30 times more expensive than low cost generics. As a result, reducing potential savings [6, 13, 17]. In addition, the rise in elderly populations across countries with greater prevalence of chronic diseases is leading to increased polypharmacy [18, 19], which coupled with improved management of chronic diseases such as increased use of statins at higher doses in patients at high risk of cardiovascular disease and greater control of blood pressure in patients with diabetes [12, 20], is increasing medicine consumption. Both situations will affect potential savings arising from the availability of generics.

Kwon and Yang [21] identified that no substitution effects were found after the entry of generics in Korea. with the utilization of both generics and originators rising substantially after generic entry. For example, the utilisation of *glimepiride* increased 22-fold after the introduction of generics [21]. Consequently, the off-patent market in South Korea tends to expand upon the introduction of generic drugs without any apparent substitution effect [21].

Particularly in Korea, pricing policies have traditionally focused on price cuts, under the assumption that lowering prices would directly curtail drug expenditures. However, the launch of an appreciable number of cheaper generics resulted in an increase, rather than a decrease, in drug expenditures by expanding the market along with an appreciable increase in their utilization. Kwon et al. [22] identified that the market expansion of antihyperlipidemic medicines in recent years was mainly attributed to the increase in the number of patients prescribed atorvastatin following patent expiration. Other studies also demonstrated that the quantity of statins prescribed significantly increased in Korea following patent expiration, especially atorvastatin [23, 24]. When faced with such findings indicating that the introduction of less costly generics contributes to increasing drug spending in Korea, and potentially other countries, by inducing market expansion instead of bringing about expected cost savings, further studies are needed to identify which factors contribute to market expansion to guide future policies in these countries.

Consequently, this study aims to explore patterns of generic prescriptions among the statins (antihyperlipidemic agents) and subsequently examine factors contributing to the market expansion after atorvastatin patent expiration using the national health insurance (NHI) claims data in Korean context. The findings will be used to provide future guidance to the NIH and other key stakeholder groups. This builds on our previous findings with the statins [21, 22].

## Methods

### Data source and study population

This study is based on two merged datasets. One is the nationally representative dataset of 2008, which was a nationwide stratified, multistage probability sampling design used in consideration of the location and residence-type in order to establish full representativeness for the whole nation [25]. We merged this dataset with the Korean national health insurance (NHI) claims data from 2007 to 2010 using a de-identified code of each individual provided by the Health Insurance Review and Assessment Agency.

As a result, the analysis of the dataset used in this study was based on the medical service utilization records of a nationwide representative sample population. The merged Korean NHI claims data comprises the medical service utilization data of the sample population classified into treatment data, prescription data, and illness data. The prescription data carries information on the date of the prescription, the name of the prescribed medicine, its dose and duration of medication, its purchase price, disease code, and type of medical institution. Details of the disease for which medication is prescribed are described in the 10^th^ revision of the International Statistical Classification (ICD-10). However, since NHI claims data does not provide laboratory data for diagnosis, diagnostic accuracy cannot be verified. This limitation often results in inaccuracies in estimations of the prevalence and incidence of diseases. Despite this disadvantage, NHI claims data have a great advantage of being a complete dataset, allowing the tracking of medical service utilization patterns of the entire Korean population.

Our study population was extracted from the dataset that covers the observation period of two years between July 2008 (patent expiration of atorvastatin) and June 2010. Included in the analysis were the data related to drug prescriptions (6,993 cases, 747 patients).

In this study, all episodes of statin prescriptions claimed during July 2008 to June 2010 (24 months) were extracted from the dataset since the patent of atorvastatin expired in July 2008 (6993 episodes, 747 persons). Statins included simvastatin (C10AA01), lovastatin (C10AA02), pravastatin (C10AA03), fluvastatin (C10AA04), atorvastatin (C10AA05), rosuvastatin (C10AA07) and pitavastatin (C10AA08). Patient’s ID was de-identified, and under each drug prescription episodes, relevant data were derived. This included drug costs, quantity prescribed, visit dates, and prescribed days. Quantities prescribed were measured in daily defined doses (DDDs) [26].

Patients were subsequently stratified by presence of statin claims (previously treated group) or absence of a statin claim (newly treated group) in the pre-index period to analyze the cohorts separately. It was hypothesized that there could potentially be differences in behavior between these two groups, based on the previous use of statins and previous publications [22, 24]. Patients where the interval from the last claim in the pre-index period to the first claim in the index period was more than 200 days (average interval = 525.25 ± 230.97 days), were considered as newly treated.

### Statistical analysis

Firstly, the descriptive time trend of prescriptions was conducted in order to identify prescribing trends after generic atorvastatin became available. In addition, the prescription quantity and time intervals between visits for prescriptions were compared according to the type of prescribed medicine, i.e. generic atorvastatin, originator atorvastatin and other statins. The coverage ratio, as calculated by dividing the quantity prescribed per each episode by time intervals, were also added for comparison. Means were tested for variance and compared by independent *t*-test, and proportions were compared using the *χ*^2^-test. All comparisons were two-sided and performed at a 5 % level of significance.

Secondly, to identify factors contributing to generic atorvastatin prescriptions, we used a Mixed Logit Model with autocorrelation error in consideration of the repeated prescription episodes among the study sample. The dependent variable with a binary structure is coded as 1 for the generic atorvastatin prescription and 0 otherwise. To control the correlations among the repeated prescription episode for the same patient sampled randomly, the first-order autoregressive (AR(1)) was modeled in covariance structure [27, 28].

The explanatory variables included demographic and socioeconomic characteristics (e.g. sex, age, education, insurance type), clinical characteristics, i.e. newly treated group or not and the number of comorbidities counting the number of 3-digit ICD for each episode, and provider characteristics, i.e. type of hospital, specialty. Medical institutions in Korea can be classified into five types: tertiary general hospitals (teaching hospitals, designated by the Minister of Health and Welfare), general hospitals (more than 100 beds), hospitals (more than 30 beds), clinics, and others that include heath centers and health care posts,

All statistical tests were performed using SAS version 9.4. PROC GLIMMIX was applied to fit our bivariate model [29]. Ethical approval was not needed since all patient data was anonymised. This is similar to other studies of this nature undertaken with anonymized health insurance company data, such as those conducted in Europe and the US [6, 12, 13, 17].

## Results

### Population characteristics

Table 1 presents the basic characteristics of the 747 patients that were prescribed statins at least once during the 24 months (July 2008–June 2010) after generic atorvastatin became available. Male patients accounted for 35.2 %, mean age was 61.62 years, and 93.1 % were NHI subscribers. Among the health care institutions, clinics occupied the highest proportion (56.7 %). The average number of outpatient visits of the subjects during the observation period of 24 months was 9.34.

**Table 1:** Basic characteristics of the study population (*n* = 747)

	Total Sample	Previously treated	Newly treated	*P*-value
	(*N* = 747)	(*N* = 283)	(*N* = 464)	
*Sex (male, %)*	35.2	34.6	35.5	NS
*Age (years), mean(SD)*	61.62(11.06)	62.96(9.76)	60.84(11.72)	0.0078
*Age group, N(%)*				
Less than 50	99(13.2)	26(9.2)	73(15.7)	
50 ~ 59	209(28.0)	82(29.0)	127(27.4)	
60 ~ 69	244(32.7)	112(39.6)	132(28.5)	
70+	135(26.1)	63(22.3)	132(28.5)	
*Insurance status, N(%)*				NS
NHI	698(93.1)	259(91.5)	439(94.6)	
Medical Aid	49(6.6)	24(8.5)	25(5.4)	
*Education, N(%)*				NS
Before or Primary school	112(15.4)	36(13.0)	76(16.8)	
Middle school	261(35.8)	105(38.0)	156(34.5)	
High school	133(18.2)	52(18.8)	81(17.9)	
College +	223(30.6)	83(30.1)	140(30.9)	
*Visits by type of institutions, N(%)* ^*a*^				
Tertiary general hospital	126(16.8)	24(16.1)	42(13.5)	0.0375
General hospital	180(24.1)	47(31.5)	61(13.2)	NS
Hospital	76(10.2)	9(6.0)	46(14.7)	0.0073
Clinics	424(56.7)	163(57.6)	261(56.1)	NS
Others	54(7.2)	12(4.2)	42(9.0)	0.0141
*Major comorbidities, N(%)*				
Hypertension	534(71.5)	245(86.6)	289(62.3)	<0.0001
Diabetes	345(46.2)	167(59.0)	178(38.4)	<0.0001
Stroke	135(18.1)	59(20.9)	76(16.4)	NS
Depression	68(9.1)	30(10.6)	38(8.2)	NS
Osteoporosis	120(17.5)	61(21.6)	59(12.7)	0.0014
*No. of visits per users during observational period*				
	9.34(8.37)	13.95(9.36)	6.55(6.23)	<0.0001

^a^Percent may be over 100 %

When comparing the basic characteristics of the previously treated and newly treated groups, significant differences were observed in the mean age, type of the health care institutions, comorbidities, and the frequency of visits for treatment. The newly treated group had a slightly lower mean age than the previously treated group (*p* = 0.0078), used tertiary general hospitals less frequently (*p* = 0.0375) while using hospitals (*p* = 0.0073) more frequently, and showed lower rates of comorbidities such as hypertension, diabetes, and osteoporosis. Newly treated users visited health care institutes significantly less frequently than previously treated users did (6.55 vs. 13.95 times) (*p* < 0.0001). Collating this, compared with previously treated users, newly treated users were generally younger, had fewer comorbidities and use health services less frequently.

### Patterns of statins prescriptions

#### Overall trend in statin utilization

As shown in Table 2, overall monthly aggregate drug spending and utilization showed an increasing trend overtime. Spending on statins and volume prescribed have increased by 36.2 % and 52.7 % respectively between July 2008 and June 2010.

**Table 2:** Monthly aggregate spending and volume prescribed for statins

	Monthly spending		Quantities	
	KRW	Δ	DDDs	Δ
July 2008	7,133,610	-	5,798	-
June 2009	8,768,838	22.9 %	7,626	31.5 %
June 2010	9,714,075	36.2 %	8,857	52.7 %

Δ: Growth rate based on July 2008

KRW: Korean Won [1 USD=1,018.71 KRW as of July 2008]

**Fig. 1:**
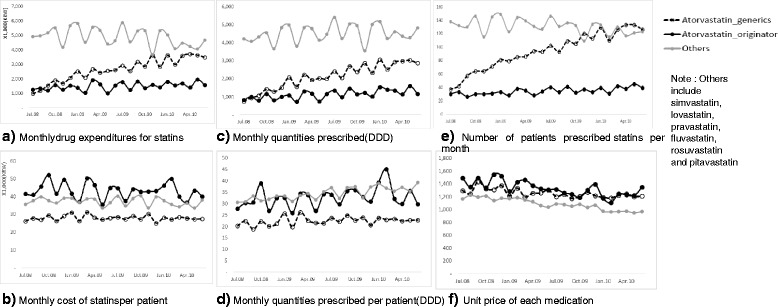
Overall trend in drug utilization and number of patients prescribed after atorvastatin patent-off

Figure 1 presents the prescription pattern of generic atorvastatin during the observation period. Monthly drug spending on generic atorvastatin sharply increased compared to its originator (see Fig. 1a). Monthly costs for generic atorvastatin were lower than the originator (Fig 1b) driven by lower quantities prescribed (Fig. 1d) since the average price for generic atorvastatin was similar to that of originator atorvastatin (Fig. 1f). With respect to utilization, there was an appreciable growth in the prescribing of generic atorvastatin versus originator atorvastatin and other statins (lower, similar or marginal increase over time) (Fig. 1c). This was driven by increasing number of patients receiving generic atorvastatin (Fig. 1e) with similar monthly quantities prescribed per patient (Fig. 1d). However, the prescribed dose for generic atorvastatin was lower than the originator (Fig. 1d).

#### Increase in number of patients prescribed statins per month

**Fig. 2:**
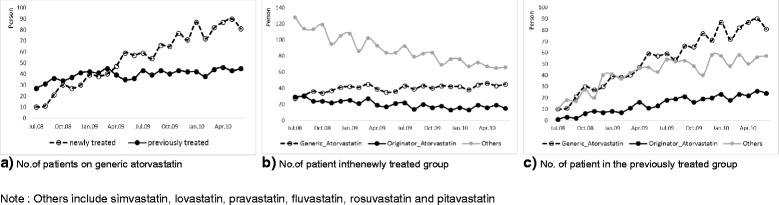
Number of patients prescribed overtime

As shown in Fig. 1e, there was a substantial increase in number of patients prescribed generic atorvastatin (240.5 % increase from July 2008 to June 2010) as opposed to the originator (30.0 %) and other statins (−10.9 %). When the patients prescribed generic atorvastatin are divided into previously treated group and newly treated group, those prescribed generic atorvastatin increasingly belong to the newly treated group (Fig. 2a).

In other words, the entry of generics after the patent expiration of atorvastatin led to a rapid increase in the number of newly treated patients, with an appreciable number prescribed generic atorvastatin (50.9 % in June 2010). Breaking down the number of the newly treated group by prescribed drug, there was increasing utilization of all statins; however, the number of the patients prescribed generic atorvastatin increased more rapidly than the other statins (Fig. 2b). This is a clear indication of the market expansion effect of generics in South Korea. Figure 2c shows the substitution effect of generic atorvastatin in the previously treated group at the expense of both originator atorvastatin and ‘other statins’ with a greater effect on ‘other statins’. While the number of patients prescribed generic atorvastatin tended to increase over time, the number of patients prescribed originator atorvastatin and other statins decreased, ‘other statins’ to a greater extent. This implies both originator atorvastatin and other statins are being substituted by generic atorvastatin to some extent in the previously treated group.

#### Prescribing pattern of generic atorvastatin

In Fig. 1d, it was verified that the prescription quantity of generic atorvastatin is lower than that of the originator atorvastatin or other statins. Table 3 presents the results of the detailed analysis of the prescription patterns of generic atorvastatin in comparison with those of the originator atorvastatin or other statins.

**Table 3:** Mean comparison of prescriptions episodes (N=6,339)

	Quantities prescribed (DDDs)	Intervals	Coverage ratio
*Prescribed medications*			
Generic Atorvastatin	20.04 ± 15.21***	38.77 ± 38.11***	0.64 ± 1.82**
Originator Atorvastatin	29.80 ± 27.48	48.65 ± 40.00*	0.91 ± 3.47
Other statins	30.59 ± 27.32	44.88 ± 44.19	0.79 ± 1.15
*Previously treated*			
Generic Atorvastatin	21.20 ± 15.65***	37.93 ± 31.99***	0.72 ± 2.57
Originator Atorvastatin	32.84 ± 31.71	53.19 ± 39.80***	0.92 ± 4.13
Other statins	27.17 ± 23.54	45.45 ± 50.05	0.74 ± 0.61
*Newly treated*			
Generic Atorvastatin	19.22 ± 14.83***	39.41 ± 42.21*	0.58 ± 0.89***
Originator Atorvastatin	25.67 ± 19.66	41.76 ± 39.35	0.89 ± 2.13
Other statins	27.17 ± 23.54	43.72 ± 51.37	0.91 ± 1.85

Note: Data on other statins were used as the reference data in the statistical analysis

*p < 0.5, **p < 0.001, ***p < 0.0001

First, the mean prescribed quantity in DDDs of generic atorvastatin is 20.04, which is significantly lower when compared with other groups (*p* = <0.0001), whereas originator atorvastatin and other statins did not show any significant differences.

The intergroup comparison of average prescription quantities yielded the following findings. Firstly, the previously treated group was prescribed generic atorvastatin at a significantly lower quantity compared to the prescription quantities of other originator atorvastatin and the other statins (*p* < 0.0001) (Table 3), whereas no differences in prescription quantities were observed between the originator atorvastatin and the other statins. The same pattern was exhibited in the newly treated group. Overall then, the prescription quantity of generic atorvastatin was significantly lower than other statins including originator atorvastatin.

However, since the prescription quantity depends on the interval between prescription refills, it is essential to consider prescription interval additionally. The mean interval for seeing a physician for prescription renewal was significantly lower in the case of generic atorvastatin (38.77 days; *p* < 0.0001), whereas original atorvastatin and other statins did not show any significant differences. Comparison of the previously treated and newly treated groups yielded results slightly different from the quantities prescribed. To find out the reason for this discrepancy, we calculated the coverage ratio by dividing the prescribed quantity at time of visiting a physician for prescription renewal by the interval, the span of time for its consumption. The coverage ratio represents the medication adherence for the prescribed quantities in the span of time till the next visit to physician for prescription renewal. A coverage ratio lower than 1 suggests that the patient missed at least 1 dose of medication. According to the coverage ratio in Table 3, there was underutilization of generic atorvastatin prescriptions among newly treated patients. The coverage ratio for those episodes is 0.58 which was significantly lower than the others (*p* < 0.0001).

### Factors contributing to generic atorvastatin prescriptions

Table 4 presents the results of the mixed logistic model based on the AR(1) covariance matrix for the factors contributing to the prescription of generic atorvastatin after the patent expiration of its branded version.

As mentioned, we considered the mixed logit model adjusting demographic and socioeconomic factors, clinical factors and provider’s characteristics. Demographic and socioeconomic status were not significantly associated with generic atorvastatin prescriptions. Only the age group of 60 ~ 69 were more likely to be prescribed generic atorvastatin [OR = 1.42; CI: 1.01–2.00]. The greater number of comorbidities, the less likely patients will be prescribed generic atorvastatin [OR = 0.96; CI: 0.94–0.98]. Newly treated patients were 2.59 times more likely to receive prescriptions for generic atorvastatin than previously treated patients [CI: 2.07–3.23] (Table 4).

**Table 4:** Mixed logit model results on factors contributing to generic atorvastatin prescription

Parameter		Estimates	SE	Odds Ratio [95 % CI]
Intercept		– 3.059***	0.356	0.05 [0.03: 0.07]
Sex (ref = female)	Male	– 0.080	0.131	0.92 [0.71: 1.19]
Age (ref = < 50)	50 ~ 59	– 0.189	0.164	0.83 [0.60: 1.15]
	60 ~ 69	0.351*	0.172	1.42 [1.01: 2.00]
	70+	0.279	0.184	1.32 [0.92: 1.91]
Education (ref: < = primary)	Middle school	0.165	0.168	1.18 [0.85: 1.64]
	High school	0.002	0.201	1.00 [0.68: 1.49]
	College +	0.233	0.199	1.26 [0.86: 1.87]
Insurance type (ref = medical aid)	NHI	– 0.027	0.189	0.97 [0.53: 1.77]
Number of comorbidities		– 0.044***	0.010	0.96 [0.94: 0.98]
Newly treated group (ref = previously treated)		0.951***	0.003	2.59 [2.07: 3.23]
Type of Hospital (ref = teaching hospitals)	General hospitals	1.378***	0.213	3.97 [2.60: 6.06]
	Hospitals	2.223***	0.229	9.23 [5.86: 14.56]
	Clinics	2.283***	0.201	9.81 [6.58: 14.61]
Specialty (ref = others)	Intemalist	– 0.057	0.076	0.95 [0.81: 1.10]
AR(1)		0.729***	0.008	
Residual		1.021***	0.029	
–2 log Likelihood		25525.50		

*p < 0.1, ***p < 0.0001

AR(1): first order auto-regressive process

When comparing generic atorvastatin prescriptions among healthcare providers by type and their specialty, then taking a tertiary general hospital as reference, the odds ratio was 3.97 [CI: 2.60–6.06] for a general hospital, 9.23 [CI: 5.86–14.56] for a secondary hospital, and 9.81 [CI: 6.58–14.61] for a clinic. From this finding, it can be inferred that secondary hospitals and clinics prescribe generic atorvastatin more frequently than tertiary hospitals, which reached statistical significance. Physician specialties made little difference. In summary, the availability of generic atorvastatin resulted in an increase in the number of prescriptions for statins, mostly through the newly treated group, and clinics and secondary hospitals are key drivers of generic atorvastatin prescriptions.

## Discussion

In this study, we firstly identified market expansion induced by newly entered generic atorvastatin in South Korea. Specifically, a substantial proportion of generic atorvastatin prescriptions were dispensed to the newly treated group and the increase in the number of newly treated patients was primarily responsible for the increase in the utilization of generic atorvastatin while the market entry of generic atorvastatin has a partial substitution effect in the previously treated group. Thus, the market expansion effect of the market entry of generic atorvastatin can be explained by the increase in the number of newly treated patients receiving prescriptions for generic atorvastatin.

Furthermore, we investigated the factors surrounding the prescribing of generic atorvastatin once it entered the market place as well as the prescribing patterns of the other statins including originator atorvastatin. The findings showed that the number of patients prescribed generic atorvastatin rapidly increased and that the newly treated group of patients was mostly responsible for this increase. We also showed that the average prescribed dose of generic atorvastatin was significantly lower in the newly treated group compared to originator atorvastatin or other statins and that clinics and secondary hospitals were key players implicated in boosting the prescription of the generic versions. Further research will focus on the rationale for this since such prescribing habits need to be addressed to ensure the full health gain from prescribing statins is accrued in these patients.

As mentioned, in many countries there is an increasing trend is to promote the utilization of lower-priced generics versus originators and patented products in a class where all products are seen as therapeutically equivalents. such as the statins, to contain healthcare costs in the face of continuing resource pressures [6, 12–14, 16, 17, 30–32]. In this study, we could verify two effects generated by the entry of generic medicines in Korea: substitution effect and market expansion effect. While the substitution effect was demonstrated in the previously treated group, the market expansion effect was observed in the increase in the utilization of statins in the newly treated group especially generic atorvastatin (Fig. 2a and b).

In Korea, given that there is no marked price difference between originals and generics and no specific demand-side measures encouraging the use of generics, changes in the overall market structure caused by the entry of new generic drugs are of particular interest. Furthermore, in the face of the situation in which the entry of generic medicines appears to act as a driving force for increasing rather than for reducing drug spending, policies regarding generic drugs will have to be reconsidered in Korea alongside the appropriateness of prescribing.

One of the limitations of this study is that we did not explore the rationale behind the considerable increase in the number of newly treated persons (Fig. 2b). This is partly attributable to the nature of the NHI claims data lacking laboratory data or diagnosis information, unlike in other countries, which makes it difficult to identify accurate diagnosis including ICD-10 data. For the same reason, the characteristics of new patients could not be determined more clearly by adjusting the rapidly increased number of new patients with respect to any increases in cases of hyperlipidemia. This will have to be addressed in a future study once epidemiological data on hyperlipidemia are secured alongside research among physicians to ascertain their rational for any rapidly increasing prescribing of statins following the availability of generic atorvastatin. This can include increased awareness of the need to treat patients well with established CV disease to reduce subsequent morbidity and mortality, as seen in the UK with their multiple initiatives [12].

One potential rationale behind the rapid increase in the number of patients prescribed statins in the newly treated group may be partly explained by the physician-induced demand in Korea as seen in a study conducted by Kim [33], i.e. increase in the number of patients initially diagnosed in clinics following greater promotion and marketing activities among pharmaceutical companies, thus contributing to the overall increase in the healthcare costs and patients’ visits. In other words, physician-induced demand can be considered partly responsible for the appreciable increase in number of new patients prescribed statins (Fig. 2b).

In addition, previous studies on physicians’ prescribing behaviors identified financial incentives associated with increased prescribing. It has been seen in a number of studies that the size of mark-up and rebates [34–38] as well as gifts offered by pharmaceutical manufacturers [39–45] greatly influenced prescription patterns. Korean domestic pharmaceutical companies are more focused on generics than newly developed medicines [46], and the patent expiration of a high volume medicine such as atorvastatin can give rise to a simultaneous release of over 100 branded generic medicines [47]. However, this strong competition among generic manufacturers is not associated with lower prices as seen in a number of other countries where prices of generic statins can be as low as 2 % to 4 % of pre-patent loss prices with market competition [12, 17, 48]. As a result, leading to generic manufacturers pursuing illegal promotional activities, such as providing physicians kickbacks for prescribing their particular products in view of the margins involved. Physicians received kickbacks of up to 20 % of the value of the prescription [49]. Under these circumstances, it may be inferred that the maximization of financial incentives through higher prescription performance is sought by creating new patients for prescription episodes whilst encouraging the prescribing low dose generic statins to minimize side effects, although evidence for this hypothesis has yet to be established. Ways to address this include greater education of physicians regarding optimal doses of statins prescribed as well as encouraging International non-proprietary name (INN) prescribing as opposed to the prescribing of branded generics as seen in some European countries [12]. This would reduce the incentive of different generic manufacturers to appreciably increase the market after patent expiry through their promotional activities. In addition, appreciable price decreases could be achieved if pharmacists were only reimbursed the price of lower priced generics with manufacturers striving to achieve this as seen for instance in the United Kingdom [12]. INN prescribing also reduces patient confusion if they are dispensed different branded generics without a full explanation, potentially leading to either under- or over-dosing [50].

## Conclusion

In conclusion, we believe this study appreciably adds to the literature in that it identified current prescription patterns characterized by a substantial increase in the prescribing of generics in new patients in Korea following their entry, the minor substitution effect on previously prescribed patients as well as the major market expansion effect generated by the rapid increase in the number of new patients prescribed generics. In follow-up studies, other issues related to the entry of generic drugs will be addressed, such as physician-induced demand, the quality of prescribing and rational use of drugs.

## Availability of data and materials

Data used for this study can be purchased by making a requeste to Health Insurance Review & Assessment Agency - however only for academic research.
