# Erratum to: Do newly marketed generic medicines expand markets using descriptive time series analysis and mixed logit models? Korea as an exemplar and its implications

**DOI:** 10.1186/s12913-017-2114-6

**Published:** 2017-03-15

**Authors:** Hye-Young Kwon, Brian Godman

**Affiliations:** 1 Division of Biomedicine & Public Health, Mokwon University, Daejeon, 35349 South Korea; 2 Division of Clinical Pharmacology, Department of Laboratory Medicine, Karolinska Institute, Karolinska University Hospital Huddinge, Stockholm, SE-141 86 Sweden; 3 Strathclyde Institute of Pharmacy and Biomedical Sciences, University of Strathclyde, Glasgow, G4 0RE UK

## Erratum

Following the publication of this article [1], it was brought to our attention that Fig. 2 contains an error: the legends for graphs b and c are interchanged.

The corrected Fig. 2 is provided below:

**Fig. 2:**
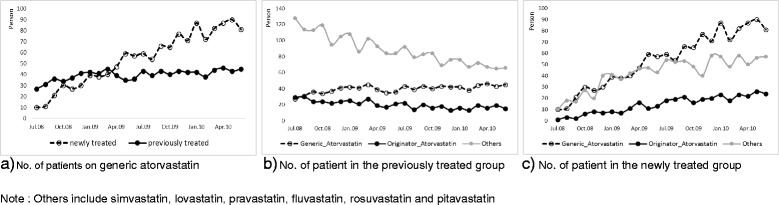
Number of patients prescribed overtime
